# Work satisfaction of professional nurses in South Africa: a comparative analysis of the public and private sectors

**DOI:** 10.1186/1478-4491-7-15

**Published:** 2009-02-20

**Authors:** Rubin Pillay

**Affiliations:** 1 School of Business and Finance, University of Western Cape, Bellville, Cape Town, South Africa

## Abstract

**Background:**

Work satisfaction of nurses is important, as there is sufficient empirical evidence to show that it tends to affect individual, organizational and greater health and social outcomes. Although there have been several studies of job satisfaction among nurses in South Africa, these are limited because they relate to studies of individual organizations or regions, use small samples or are dated. This paper presents a national study that compares and contrasts satisfaction levels of nurses in both public and private sectors.

**Methods:**

This was a cross-sectional survey of professional nurses conducted throughout South Africa using a pretested and self-administered questionnaire. Univariate and bivariate statistical models were used to evaluate levels of satisfaction with various facets of work and to elicit the differences in satisfaction levels between different groups of nurses. A total of 569 professional nurses participated in the study.

**Results:**

Private-sector nurses were generally satisfied, while public-sector nurses were generally dissatisfied. Public-sector nurses were most dissatisfied with their pay, the workload and the resources available to them. They were satisfied only with the social context of the work. Private-sector nurses were dissatisfied only with their pay and career development opportunities. Professional nurses in the more rural provinces, those intending to change sectors and those more likely not to be in their current positions within the next five years were also more likely to be dissatisfied with all facets of their work.

**Conclusion:**

This study highlighted the overall dissatisfaction among South African nurses and confirmed the disparity between the levels of job satisfaction between the public and private sectors. Health managers should address those factors that affect job satisfaction, and therefore retention, of nurses in South Africa. Improving the work environment so that it provides a context congruent with the aspirations and values systems of nurses is more likely to increase the satisfaction of nurses and consequently have a positive effect on individual, organizational and health outcomes.

## Background

South Africa has a dual health system. The public sector, comprising government health institutions, serves predominantly the indigent population, while the private sector, comprising for-profit organizations and individuals, serves the insured population or those who can afford care on an out-of-pocket basis. Although the public sector is responsible for the well-being of 82% of the population, it accounts for only 40% of the total health expenditure in South Africa. In contrast, the private sector consumes 60% of the health expenditure and is responsible for less than 20% of the population [1]. The public sector, which is underresourced and overused, is often characterized as being inefficient and ineffective in terms of meeting its mandate of accessible, affordable and appropriate health care. The private sector, on the other hand, is reputed for its world-class facilities and care provision.

Given the pivotal role that nurses play in determining the efficiency, effectiveness and sustainability of health care systems, it is important to understand what motivates them and the extent to which the organization and other contextual variables satisfy them. Job dissatisfaction has frequently been cited as the primary reason for a high turnover of nurses [2-5], as well as increased rates of absenteeism [6], both of which impede efficiency and effectiveness, which in turn pose a threat to a health care organization's capacity to provide good care as well as meet the needs of patients [7,8]. This may result in a decrease in morale and productivity of the remaining nurses due to the increasing pressure on them [9], which in turn has the potential to contribute to further work dissatisfaction and a further increase in nurse turnover [10]. In addition, work satisfaction was found to be an important predictor of where health professionals intended to work [11,12].

Work satisfaction is also an essential part of ensuring high-quality care. Dissatisfied providers not only give poor quality, less efficient care [13], there is also evidence of a positive correlation between professional satisfaction and patient satisfaction and outcomes [3,14-16]. Nurses who were not satisfied at work were also found to distance themselves from their patients and their nursing chores, resulting in suboptimal quality of care [17]. Baxter [18] further highlighted the strong influence of nurse job satisfaction on the quality of care they provided as well as on the nurse-patient relationship [19].

There is also compelling evidence of a positive relationship between job satisfaction and employee health. Blegen et al. [20] demonstrated a strong negative association between work satisfaction and stress, while Grieshaber et al. [21] showed that dissatisfaction led to increased stress and frustration, which resulted in physical, emotional and behavioral problems. This has been found to be an important contributor to suboptimal performance of nurses [22] as well as to abandonment of the profession [23].

Job satisfaction has primarily been defined by two approaches: a global approach that encompasses overall attitudes, feelings and emotions towards their work experience [24,25], and a faceted approach that emphasizes employees' attitudes towards individual aspects of their job [26,25], which is more useful at determining specific areas for improvement [27].

Although international research varies in its specific findings, the general conclusions seem to support a sentiment of growing dissatisfaction experienced by nurses around the world [28-32]. Key dissatisfactors were found to include non-supportive work environments [33] and increased workloads [34,35], while important predictors of nurse work satisfaction included autonomy [36,37], work content [38], professional development and recognition [39], and relationships with co-workers and peers [40]. Although the literature on the subject demonstrates areas of commonality, it also highlights significant differences between different labour markets [26].

In South Africa, nurses were also found to be generally dissatisfied [41,42], with remuneration being a key contributor to dissatisfaction [42-45]. Poor working conditions and organizational climate were also strong predictors of dissatisfaction [43,46], while the social context of the job was found to be a strong predictor of satisfaction [43,45,47]. The South African studies are, however, limited in the sense that they relate to studies in individual organizations [43], are done at regional level [46,45], used small samples [46], or used data collected prior to the sociopolitical transition and health system transformation [41,42]. There have been no recent studies of this phenomenon nationally, and none have compared and contrasted work satisfaction levels of nurses in the different sectors. This paper is an attempt to address this gap in the literature.

## Methods

Data for this study came from a survey of professional nurses throughout South Africa that formed part of a broader study of attraction, work satisfaction and retention issues. Based on a population of 99 534 professional nurses registered with the South African Nurses Council, the minimum sample size was calculated at 367, at a 95% level of certainty and allowing for a margin of error of 5%. Based on a response rate of around 40% for similar studies [11], a computer-generated simple random sample of 1000 professional nurses was selected. A self -administered questionnaire that had been specially developed and pretested was posted to nurses in September 2006 and non-responders were sent questionnaires up to two more times at intervals of four weeks. Data collection was terminated six weeks after the final mailing. The results of a sample of primary non-responders (non-respondents to the first mailing attempt) were compared to that of the primary responders (respondents to the first mailing attempt) to assess non-response bias [48].

The objective of this study was to determine the extent of work satisfaction among nurses and to examine variables influencing 13 aspects of job satisfaction. Scales combining multiple items were used to measure satisfaction with autonomy, relationship with nursing colleagues, patient care, relationship with doctors, personal time, relationship with management, career opportunities, safety, the community, pay, resources, workload and satisfaction with their careers. Some of the items and scales were developed by the researcher, while others were adapted from the literature [3,27,49-51].

Factor analysis using principal components analysis was used to evaluate the construct validity of the satisfaction subscales and the Kaiser's eigenvalue rule was used to determine the factors to be extracted from the 58 satisfaction items. Satisfaction was measured by means of a five-item Likert scale ranging from 1 (very dissatisfied) to 5 (very satisfied). The reliability of the factors was estimated by assessing the internal consistency of the scales by means of Cronbach's alpha. The ultimate score that each nurse received for each factor was based on the simple summation of the item scores for that measure. Mean factor scores at the lower end of the score range denote lower satisfaction with a particular facet, while higher scores denote satisfaction.

The independent variables in the study were the respondent's personal characteristics, work experience and future work plans. One-way analysis of variance (ANOVA) was used to test the statistical significance of the differences in work satisfaction between nurses in the public and private sectors and other categorical variables, while the chi-square test was used to test the statistical significance of the association between categorical variables.

## Results

After the primary and two follow-up surveys, questionnaires were returned from 569 of 907 valid addresses of professional nurses. This represents a total response rate of 62.7%. There were no significant differences between primary respondents (297) and the sample of primary non-respondents in terms of demographics, future work plans and satisfaction levels.

As shown in Table 1, most respondents were female (94.1%), above the age of 40 (73.1%) and had more than 20 years' experience (60.4%). Most of the respondents were from Gauteng (26.8%), Kwa-Zulu Natal (20.8%) and the Western Cape (16%), while Mpumalanga (4.8%), North West (4.3%) and Northern Cape (2%) had the fewest respondents.

**Table 1:** Respondent characteristics and future work plans

		**Frequency**	**Valid percentage**
Gender (N = 559)	Male	33	5.9

	Female	526	94.1

	Total	559	100

Age (N = 562)	< 30	24	4.3

	30–40	127	22.6

	> 40	411	73.1

	**Total**	**562**	**100**

Place of employment (N = 557)	Mpumalanga	27	4.8

	Limpopo	42	7.5

	Northern Cape	11	2

	Gauteng	149	26.8

	Free State	36	6.5

	North West	24	4.3

	Eastern Cape	63	11.3

	Kwa-Zulu Natal	116	20.8

	Western Cape	89	16

	**Total**	**557**	**100**

Sector of employment (N = 550)	Private	219	39.8

	Public	331	60.2

	**Total**	**550**	**100**

Intention to change sector (N = 540)	Yes	188	34.8

	No	352	65.2

	**Total**	**540**	**100**

Years of nursing experience (N = 563)	0–9	66	11.7

	10–19	157	27.9

	> 20	340	60.4

	**Total**	**563**	**100**

Work plan for next 5 years (N = 536)	Remain in current position	162	30.2

	Leaving nurse for another profession	68	12.7

	Quit nursing and retire from all forms of employment	50	9.3

	Work abroad	84	15.7

	Further training in nursing	125	23.3

	Others	47	8.8

	**Total**	**536**	**100**

About 40% of respondents worked in the private sector, while 60% worked in the public sector. Some 34.8% of respondents indicated an intention to change their sector of employment within the next five years, while 30.2% reported that they would most likely still be in their current positions as professional nurses in five years' time.

Factor analysis yielded the same 13 facets that captured 76% of the variance in nurses' responses to the satisfaction items. Cronbach's alpha–a measure of internal consistency–was well within acceptable range for all scales (α > 0.70).

Overall, professional nurses in South Africa were marginally dissatisfied (mean 2.935) (Table 2). They did, however, express greatest satisfaction in their relationship with patients and the gratification they obtained from patient care (3.734), their relationship with their nursing colleagues (3,582), doctors (3.391) and their sense of belonging in the communities within which they work (3.368). They were most dissatisfied with their pay (2.020), the workload (2.244), their career development opportunities (2.595) and the resources available to them (2.727).

**Table 2:** Reliability and descriptive statistics for work satisfaction subscales

**Satisfaction**	**All respondents**			**Private sector**			**Public sector**		
Factors	N of items	Cronbach's alpha	Mean	N of items	Cronbach's alpha	Mean	N of items	Cronbach's alpha	Mean

Autonomy	4	0.869	3.059	4	0.854	3.487	4	0.85	2.803

Resources	5	0.887	2.727	5	0.848	3.397	5	0.84	2.306

Career opportunities	6	0.925	2.592	6	0.936	2.987	6	0.896	2.337

Relation with nurses	4	0.897	3.582	4	0.893	3.697	4	0.901	3.514

Patient care	5	0.878	3.734	5	0.867	3.984	5	0.872	3.571

Relation with management	5	0.96	2.679	5	0.966	3.124	5	0.943	2.406

Relation with doctors	4	0.939	3.391	4	0.941	3.528	4	0.939	3.317

Personal time	4	0.917	2.868	4	0.939	3.12	4	0.896	2.706

Safety	4	0.925	2.839	4	0.923	3.57	4	0.878	2.351

Community	3	0.902	3.368	3	0.915	3.598	3	0.887	3.226

Pay	4	0.919	2.02	4	0.934	2.397	4	0.881	1.787

My career	4	0.87	3.047	4	0.888	3.507	4	0.835	2.751

Workload	5	0.922	2.244	5	0.927	2.777	5	0.889	1.94

**Overall satisfaction**			**2.935**			**3.321**			**2.693**

Private-sector nurses were generally satisfied (3.321). They expressed greatest satisfaction with the gratification they obtained from patient care (3.984), their relationship with their nursing colleagues (3.697), their sense of belonging in the communities within which they work (3.598), safety in the work environment (3.570) and their relationship with doctors. They were dissatisfied only with their pay (2.397) and career development opportunities 2.987).

Public-sector nurses were generally dissatisfied (2.693). They did, however, express satisfaction with the gratification they obtained from patient care (3.571), their relationship with their nursing colleagues (3.514) and doctors (3.317) and their sense of belonging in the communities within which they work (3.226). They were most dissatisfied with their pay (1.787) and workload (1.940), followed by the resources available to them (2.306), career development opportunities (2.337) and safety of the work environment (2.351).

Analysis of variance showed significant differences between the satisfaction levels of nurses with different bio-demographic characteristics, from different provinces, with different levels of experience, in the different sectors, as well as those who intended to change sectors or had other work plans within the next five years (Table 3).

**Table 3:** Differences in satisfaction levels between different categories of nurses: ANOVA F-values and levels of significance (p)

**Satisfaction factors**	**Gender**	**Age**	**Province**	**Sector**	**Change sector**	**Experience**	**Work plan**	**Return**
Autonomy	0.904 (0.342)	0.449 (0.639)	1.410 (0.189)	64.985 (0.000)	48.767 (0.000)	2.272 (0.104)	17.040 (0.000)	2.810 (0.066)

Resources	3.850 (0.050)	0.022 (0.978)	3.770 (0.000)	225.148 (0.000)	17.941 (0.000)	3.005 (0.030)	19.399 (0.000)	3.378 (0.039)

Career opportunities	0.065 (0.798)	2.793 (0.062)	1.643 (0.110)	60.310 (0.000)	57.493 (0.000)	7.389 (0.001)	22.204 (0.000)	1.972 (0.146)

Relation with nurses	0.160 (0.689)	0.287 (0.751)	1.350 (0.216)	5.793 (0.016)	7.617 (0.001)	0.133 (0.941)	5.470 (0.000)	1.240 (0.295)

Patient care	1.240 (0.266)	1.911 (0.149)	0.492 (0.862)	35.100 (0.000)	12.951 (0.000)	3.297 (0.038)	8.352 (0.000)	0.747 (0.477)

Relation with management	0.047 (0.828)	2.831 (0.060)	2.579 (0.009)	58.031 (0.000)	60.079 (0.000)	6.974 (0.001)	21.720 (0.000)	0.914 (0.405)

Relation with doctors	0.546 (0.460)	4.611 (0.010)	2.062 (0.038)	5.425 (0.020)	6.131 (0.002)	3.735 (0.011)	3.901 (0.002)	0.437 (0.647)

Personal time	0.529 (0.467)	0.790 (0.455)	2.742 (0.006)	22.198 (0.000)	9.478 (0.000)	2.374 (0.069)	7.157 (0.000)	1.617 (0.207)

Safety	0.589 (0.443)	0.661 (0.517)	5.317 (0.000)	188.452 (0.000)	27.226 (0.000)	3.793 (0.010)	16.494 (0.000)	2.461 (0.092)

Community	0.049 (0.825)	1.914 (0.149)	3.245 (0.001)	19.480 (0.000)	14.037 (0.000)	3.525 (0.015)	10.518 (0.000)	0.904 (0.409)

Pay	0.452 (0.501)	1.190 (0.305)	2.239 (0.023)	56.230 (0.000)	27.994 (0.000)	2.539 (0.056)	21.161 (0.000)	1.483 (0.233)

My career	0.585 (0.445)	1.640 (0.195)	1.962 (0.049)	78.945 (0.000)	24.174 (0.000)	3.337 (0.019)	20.555 (0.000)	3.848 (0.025)

Workload	0.063 (0.803)	0.414 (0.661)	2.135 (0.031)	97.721 (0.000)	32.510 (0.000)	1.913 (0.149)	12.914 (0.000)	1.617 (0.207)

Female nurses were generally more satisfied with resources than their male colleagues (F = 3.85, p < 0.05), while nurses above age 40 were significantly more satisfied than their younger colleagues with their relationships with management (F = 2.831, p < 0.05) and with doctors (F = 4.611, p < 0.01). Nurses with more than 20 years' experience were also significantly more satisfied than their less-experienced colleagues with most of the facets of their work.

Nurses in the Western Cape, Free State, Kwa-Zulu Natal and Gauteng were significantly more satisfied than their colleagues from other provinces with most of the facets of their work except for their relationships with the community, where nurses in Limpopo and North West were more satisfied.

Nurses in the private sector were also significantly more satisfied with all facets of their work than their colleagues in the public sector. The greatest differences in satisfaction levels were with regard to safety, resources available, workload, their careers and their relationship with management, respectively

Nurses who intended to change their sector of employment were significantly less satisfied in all the work facets than those who indicated that they would not be changing their sector of employment. Nurses who intended to remain active in the profession for the next five years were also significantly more likely to report being satisfied with all the facets of their work, as opposed to those whose work plans included either quitting the profession, working abroad or pursuing further training in nursing.

## Results and discussion

Given that it is difficult to achieve desirable response rates in surveys of health professionals, the relatively high response rate of more than 60% suggests that the iterative process is appropriate in this context. It may also indicate high interest among nurses in the subject area. The similarity in response rates between respondents and a sample of primary responders suggests that non-response bias was minimal and the sample was therefore representative of all professional nurses in South Africa.

Results from the survey demonstrate that although nurses are generally dissatisfied, discrepancies are masked between levels of satisfaction with different aspects of their work, between nurses with different bio-demographic characteristics, between nurses in the different sectors and from different geographic regions, and between nurses with different future work plans.

The overall dissatisfaction among the cohort surveyed is disconcerting, given that work satisfaction is positively correlated to increased absenteeism [6] and turnover [2-5], nurse morale and health [21,20], productivity [9] and clinical outcomes [3,14-16]. This, in turn, has implications for the efficiency, effectiveness and sustainability of our health care system.

Overall, nurses in the public sector were generally dissatisfied, while nurses in the private sector were satisfied. This contradicts the general management literature, which suggests that public sector satisfaction has improved relative to the private sector over the last decades [52]. It does, however, support the nursing management literature, which demonstrates significant dissatisfaction among public-sector nurses relative to their private-sector colleagues [53], suggesting that the work milieu in the public sector does not meet the aspirations and values systems of nurses.

Public-sector nurses were most dissatisfied with their pay, workload and the resources available to them, while private-sector nurses were moderately dissatisfied with pay and workload and marginally dissatisfied with their career opportunities. Although both sectors have identified pay and workload as being an important source of dissatisfaction, private-sector nurses are relatively more satisfied with these factors than their public-sector counterparts. The question then arises: Why are private-sector nurses significantly more satisfied than their public-sector colleagues? The data suggest that nurses in both sectors were satisfied with the social context of their work (relationships with colleagues, doctors and communities) and the intrinsic satisfaction they receive from patient care. In other words, they are happy with the work they do and the people with whom they work. This is positive, given that optimal health provision depends on teamwork and interprofessional cooperation and communication.

The differences in satisfaction are related to the work context: safety, resources, workload and work schedule, management, pay and autonomy. The biggest difference in satisfaction levels was in the perceived levels of safety in the workplace–personal safety, risk of infection, risk of injury and the physical work environment. Public-sector nurses were extremely dissatisfied, while private-sector nurses were satisfied. The emergence of deadly diseases such as extreme drug-resistant tuberculosis (XDR TB) and HIV/AIDS, in the wake of the already burdened public health care system, probably contributes to the weakening of the safety of the nursing work environment. In addition, patients with these illnesses generally require more specialized care and longer-term treatment than other patients, further increasing the workload.

Andrews & Dziegielewski [54] in their study on nurses in the United States of America also highlighted nursing as a hazardous occupation, with job-related injury and illnesses among nurses being among the highest in the workforce. There is also sufficient anecdotal evidence to suggest that nurses are also frequently victims of bullying from managers and more senior colleagues and physical violence or threats of violence, often from patients or patients' relatives. Nurses are therefore being restricted from working to their full potential and providing total commitment as a result of their constrained environment. Total commitment of knowledge workers, according to Davenport [55], depends upon providing an environment that encourages adequate use of their abilities. The availability of protective materials and functional equipment to safeguard nurses from unnecessary accidents, as well the implementation of structures and processes to help nurses improve their personal safety skills and provide support for victims of workplace violence [56], will help contribute to a safer environment for nurses in the public health care setting.

The difference in satisfaction levels with resources available–working equipment, medication, examination facilities, time and staff–is also stark, with nurses working in private health care afforded the ideal opportunity to improve the health care status of patients in an optimal setting with adequate resources and time. This translates into more efficient, effective health care in a more comfortable environment, which ensures that patients become the priority and patients' needs are met.

The issue of nurse remuneration, especially in the public sector, has been an enduring one [45,47,42,41]. This may partly explain the move of so many public sector nurses to the private sector. Higher salaries offered by overseas hospitals are also proving to be an ideal pull factor. However, it is hoped that the Occupation Specific Dispensation, which significantly improved the salaries of public sector nurses and which was implemented in January 2008 and backdated to June 2007, will address this issue to some extent. Health administrators will also do well to link portions of remuneration to performance objectives such as quality of care, resource conservation and patient-centric care, as well as to consider non-financial (e.g. career development opportunities) and psychological rewards (e.g. gratitude and recognition) [57].

An increased workload for nurses–resulting from the severe shortage of nurses as well as an increase in demand for care–has been associated with burnout and intention to leave [23]. Excessive workload has been shown to significantly contribute to public- and private-sector nurses' dissatisfaction in South Africa. Tzeng [3] was able to demonstrate in her study among Taiwanese nurses that workload was a predictor of nurse turnover. This results in increased workload for the remaining nurses, which in turn decreases the morale and productivity of those who remain, further increasing turnover [9]. The implications of these findings are therefore alarming for the provision of health care in South Africa now and in the future, given that we are already facing challenges with regard to nurse retention. In addition, the long, irregular and inflexible working hours have the potential to adversely affect family dynamics. Consideration should be given to improving scheduling and providing day care for children and more part-time employment, all of which are bound to have a positive effect on the personal lives of nurses as well.

The general dissatisfaction of public-sector nurses with their careers and the career opportunities available to them is a further measure of demoralization of nurses and offers some substantiation of the disaffection associated with working in the public sector. The decreasing attractiveness of nursing as a career is of great concern, given that nurses play a central role in the government's primary health care approach. Attraction, retention and motivation difficulties may in the long run offset the gains of attempts to improve efficiency within the health delivery system. The results were marginal, but the data indicated that private-sector nurses were also dissatisfied with career opportunities available to them as nurses in South Africa. Although this finding is not supported by Barrows & Wesson [54], who found private-sector employees significantly more satisfied with their career opportunities than their public-sector counterparts, this may partly explain why private-sector nurses leave the profession or the country.

Career opportunities and training afford individuals the prospect of further developing themselves and growing within the ranks of their career. They also acknowledge experience and time dedicated to nursing, which provides much-needed recognition in the field of nursing. These findings are in line with the suggestion by Horwitz et al. [58] who proposed that highly effective strategies for motivation and retention of knowledge workers need to be centred on creating a stimulating and challenging environment. Career development was identified by Irvine & Evans [59] as contributing significantly to decreased job turnover and it is therefore a crucial management function.

The significantly different responses to the satisfaction factors by nurses intending to leave their current employment sector and those not intending to leave are confirmation of the push factors that direct the ultimate movement of nurses out of the public sector into the private sector. Responses to all factors were significantly different and the levels of satisfaction experienced by the nurses who intend to leave their current sector were significantly lower for all factors than those who intend to stay. These results support the findings of Shields & Ward [8] that work dissatisfaction is a strong predictor of intention to quit and confirm the findings of Pillay [11] that work satisfaction is an important predictor of where health professionals intended to work. They underscore the consequences of failure to address nurses' causes of dissatisfaction, which reflect the many hygiene factors [60]. This further highlights the need for government to recognize the needs of nurses and work towards improving them. Failure to do so may well result in increased migration of nurses out of the public sector and the country.

The finding that nurses in the more urbanized provinces (Western Cape, Free State, Kwa-Zulu Natal and Gauteng) were significantly more satisfied than their colleagues from the more rural provinces may also partly explain the gravitation of nurses from rural to urban areas. This further supports that assertion of Pillay [11] that:

work satisfaction is an important predictor of where health professionals intended to work. Health managers in rural provinces should therefore focus on key dissatisfactors if they are to improve retention of nurses in their regions. It was however interesting to note that that nurses in the more rural provinces were significantly more satisfied with their relationships with the communities within which they work. The role that communities can play in the recruitment and retention of nurses therefore offers us a key point of leverage to improve recruitment and retention efforts and definitely warrants more research.

The work experience of a nurse was also a significant variable that resulted in varying responses to the levels of satisfaction among the given factors. Nurses with more than 20 years' experience were more satisfied with most of the satisfaction facets than those nurses with less work experience. Years of experience bring with them a sense of security in nursing, and fewer surprises. Relationships with patients and colleagues are built and strengthened over the years that are more difficult to establish when one is less experienced. Experience also has the advantage of promoting nurses within the ranks and reserving the more menial tasks to the less experienced and younger nurses, thereby offering a manageable workload and flexible working hours. These factors are satisfying to nurses and help explain the overall higher levels of satisfaction among the more experienced nurses. This augurs well for the retention of the more experienced nurses, with the associated benefits of institutional memory retention and coaching and mentoring of new entrants.

This study had limitations that must be acknowledged. First, responders may have been more dissatisfied than non-responders, leading to exaggerated estimates of dissatisfaction. Second, the research relied on subjective assessments of respondents to the survey and it was not possible to externally validate these responses.

## Conclusion

This study highlighted the overall dissatisfaction among South African nurses and confirmed the disparity between the levels of job satisfaction between the public and private sectors. Nurses are pivotal to the effective and efficient delivery of health care in South Africa; the chronic shortages of nurses impose a real threat to its future. It therefore becomes imperative for health care managers to identify and address those factors which are the stumbling blocks to job satisfaction and therefore retention of nurses in South Africa. Improving the work environment so that it provides a context congruent with the aspirations and values systems of nurses is more likely to increase the satisfaction of nurses and consequently have a positive effect on individual, organizational and health outcomes.

## Competing interests

The author declares that they have no competing interests.
